# Ovarian Cancer Immunotherapy: Preclinical Models and Emerging Therapeutics

**DOI:** 10.3390/cancers10080244

**Published:** 2018-07-26

**Authors:** Curtis W. McCloskey, Galaxia M. Rodriguez, Kristianne J. C. Galpin, Barbara C. Vanderhyden

**Affiliations:** 1 Cancer Therapeutics Program, Ottawa Hospital Research Institute, 501 Smyth Road, Ottawa, ON K1H 8L6, Canada; cmccloskey@ohri.ca (C.W.M.); garodriguez@toh.ca (G.M.R.); kgalpin@ohri.ca (K.J.C.G.); 2 Department of Cellular and Molecular Medicine, University of Ottawa, 451 Smyth Road, Ottawa, ON K1H 8M5, Canada

**Keywords:** ovarian cancer, tumor microenvironment, immune infiltrating cells, chemotherapy, immunotherapy, syngeneic, transgenic models, hot vs. cold tumors, immunosuppression

## Abstract

Immunotherapy has emerged as one of the most promising approaches for ovarian cancer treatment. The tumor microenvironment (TME) is a key factor to consider when stimulating antitumoral responses as it consists largely of tumor promoting immunosuppressive cell types that attenuate antitumor immunity. As our understanding of the determinants of the TME composition grows, we have begun to appreciate the need to address both inter- and intra-tumor heterogeneity, mutation/neoantigen burden, immune landscape, and stromal cell contributions. The majority of immunotherapy studies in ovarian cancer have been performed using the well-characterized murine ID8 ovarian carcinoma model. Numerous other animal models of ovarian cancer exist, but have been underutilized because of their narrow initial characterizations in this context. Here, we describe animal models that may be untapped resources for the immunotherapy field because of their shared genomic alterations and histopathology with human ovarian cancer. We also shed light on the strengths and limitations of these models, and the knowledge gaps that need to be addressed to enhance the utility of preclinical models for testing novel immunotherapeutic approaches.

## 1. Introduction

At present, there are no approved immune therapies for epithelial ovarian cancer (EOC) patients. As EOC is often detected at a late stage, research has mainly focused on the discovery of new treatments. Current first-line treatment is debulking surgery and adjuvant or neoadjuvant chemotherapy. Even though >80% of patients show a positive response to this initial therapy, most patients will relapse with chemotherapy-resistant disease [1]. As the presence of tumor infiltrating lymphocytes (TILs) correlates with increased EOC patient survival [2,3,4,5,6,7,8,9], immunotherapies hold great potential for improving EOC outcomes, as they have for several other types of cancers. The U.S. Food and Drug Administration has approved the use of several immune checkpoint inhibitors for non-small-cell lung cancer (NSCLC), melanoma, bladder cancer, renal cell carcinomas, and Hodgkin lymphoma and recently approved the first chimeric antigen receptor (CAR)-T-cell therapy to treat children with B-cell acute lymphoblastic leukemia [10,11].

Antitumor immunity in EOC patients is robustly attenuated because of the immunosuppressive cells within the tumor microenvironment (TME), as reviewed in the literature [12,13]. Several cell types are found in the tumor niche, including immune cells [effector T and B lymphocytes, regulatory T and B cells, natural killer cells (NKs), tumor-associated macrophages (TAMs), and myeloid-derived suppressor cells (MDSCs), among many others (Table 1)], as well as other components found in the TME, including fibroblasts and the adipocytes in the omentum [12]. MDSCs, TAMs, and regulatory T cells (Tregs) play a critical role in maintaining a highly immunosuppressive TME by producing immunomodulatory molecules [transforming growth factor beta (TGFβ), interleukin (IL)-10, IL-6, etc.] and inducing and recruiting immunoinhibitory cells, which dampens antitumoral immunity and supports tumor promotion [12,14]. Therefore, EOC immunotherapy must combine approaches that aim to reduce the highly immunosuppressive TME, as well as stimulate immune-activating antitumoral responses. This review describes encouraging results from both preclinical and clinical trials and highlights the immunotherapies that offer innovative and combinatorial approaches to circumvent the antitumoral barriers within the TME.

## 2. Adoptive Cell Therapy

Adoptive cell therapy (ACT) aims to boost the antitumoral activity of autologous (patient) or allogeneic (from healthy donors) lymphocytes [15]. ACT consists of the isolation of T cells from a patient’s tumor or peripheral blood to expand or manipulate those cells ex vivo, away from the influence of the immunosuppressive TME. These ex vivo expanded T cells are reintroduced into the patient along with recombinant IL-2 (rIL-2) after a lympho-depleting chemotherapy regimen [15]. ACT has resulted in complete and durable regressions in patients with melanoma [16,17]. In 1995, Fujita and colleagues used ACT of TILs in 13 patients with advanced-stage EOC who did not show any detectable lesions after primary surgery and cisplatin-containing chemotherapy [18]. All patients who received TILs following post-surgery chemotherapy survived three years, compared with only 67.5% of patients receiving chemotherapy alone. Interestingly, TILs promoted tumor regression even in patients with advanced disease or recurrent platinum-resistant EOC [18]. Among TILs, CD8+ T cells have been shown to migrate and infiltrate tumors and mediate antitumoral responses [19]. An early trial in EOC patients that used ACT with TILs following a single injection of cyclophosphamide showed tumor regression in primary tumors and metastases (ovary, liver, lung, and lymph node), which was stable for up to five months with one out of seven patients showing complete response and four out of seven with >50% reduction in tumor burden [20]. When this study was expanded with 10 additional patients, seven cases showed complete regression without recurrence for up to 15 months [20]. This highlighted the prospect of combined therapy using TILs and cisplatin without rIL-2 administration that has unfavorable toxicity in many cases [20].

In another study, 11 patients with advanced platinum-resistant EOC received intraperitoneal (IP) TILs and low doses of rIL-2 IP [21]. Grade 3 clinical toxicity (peritonitis) and anemia were observed without a significant clinical response in any patient. However, 50% of treated patients had regression of ascites (two patients), tumor and carcinoma antigen (CA)-125 (one patient), and surgically confirmed stable tumor and CA-125 values (one patient) [21]. Thus, ACT efficacy has shown conflicting results depending on the study (reviewed in the literature [22]). Importantly, the most encouraging clinical responses were observed when patients were stratified according to the presence or absence of TILs, with the best clinical response in patients who had TILs or ‘hot’ tumors, a property known to improve survival [22]. As discussed by Santoiemma and Powell, these early studies were executed prior to our advanced understanding of TIL quality, persistence and specificity, patient disease burden, and pre-conditioning regimens, which have since been significantly improved [22].

As ACT has resulted in response rates up to 72% in metastatic melanoma occurring at all sites and has been durable beyond three years in many patients [17], there remains considerable promise in identifying and optimizing conditions whereby ACT can be consistently successful in EOC.

### Chimeric Antigen Receptor T Cells (CAR-T)

New approaches, such as genetically engineered T cells, build on the promising early ACT trials that are constrained by the need to isolate and expand functional tumor-reactive T cells [23]. Genetic engineering of T cells has become a powerful approach to increase tumor immunity [24]. The T-cell receptor (TCR) from lymphocytes and chimeric antigen receptors (CARs) can be adapted to specifically target patient tumor cells. CAR-T cells allow for the recognition of tumor cells in a major histocompatibility complex (MHC)-unrestricted manner, combining antigen-specificity and T-cell activating properties in a single fusion molecule [25]. The first generation of CARs was tested in several cancers, including EOC [26], renal cancer, lymphoma, and neuroblastoma, inducing modest responses [25]. In the first study of CAR-T cells in EOC, autologous T cells specific to the EOC tumor associated antigen (TAA) α-folate receptor (FRα) were generated with a chimeric gene composed of an anti-FRα single-chain antibody linked to the signaling domain of the Fc receptor gamma chain [26]. From this study, no reduction in tumor burden was seen in any patient. Although large numbers of CAR-T cells were well tolerated, they did not persist long-term [26]. Some promising TAAs related to EOC for the generation of CARs are FRα [27], human epidermal growth factor receptor 2 (HER-2) [28], CA-125 (MUC16) [29,30], and mesothelin [31]. Moreover, CAR-T cells can be redirected against NKG2D ligands, which are widely expressed on EOC [32], as well as the epithelial cell adhesion molecule (EpCAM) [33] and 5T4 [34].

CAR translation to solid tumors is actively being investigated at present; however, few facilities have the capacity to produce CARs and many trials have achieved less than expected efficacy. This could be explained by the highly immunosuppressive TME [35]. Koneru and colleagues generated T cells engineered to specifically recognize the MUC-16ecto TAA that is expressed in the majority of ovarian tumors and derived from the cleavage of CA-125 [29,36]. As a strategy to overcome the TME, they developed a construct that co-expressed both MUC16ecto CAR and IL-12, a pro-inflammatory cytokine that has potential roles in anticancer therapy [37]. Some immune cells such as NK cells, dendritic cells (DCs), and macrophages normally produce IL-12 to induce T-cell proliferation and inhibit Tregs. The IL-12 secreting CAR-T cells displayed enhanced antitumor efficacy as determined by increased survival, prolonged persistence of T cells, and higher systemic interferon gamma (IFNγ) in mice with human EOC xenografts [36]. These observations suggest that IP delivery of CAR-T cells may be most beneficial for EOC treatment [38]. The peritoneal cavity is the main locus for EOC metastases, and local treatment seems to be a safer option for patients, because adverse reactions induced by ‘on-target off-tumor’ toxicities, such as cytokine release syndrome, were reported in a study that used ACT of autologous mesothelin-redirected CAR-T cells in a patient with BRCA1+ advanced recurrent serous EOC [39]. By local administration of CARs, antigens expressed by both EOC and healthy tissue, such as EpCAM, can be targeted in a safer way [40]. Indeed, there is a long-term survival advantage associated with IP chemotherapy in advanced EOC disease [41]. Recent studies have shown that CAR-T cells could be administrated along with cytokines such as IL-2, IL-7, IL-15, and IL-21 to increase their efficacy against hematologic and solid tumors [42].

There are still many barriers to overcome for CAR-T therapy effectiveness, such as T-cell trafficking into the tumor niche, patient selection, cancer-specific TAAs, and the highly immunosuppressive TME, as well as the dose and route of administration [35,43,44]. Combinatorial strategies to circumvent these barriers, such as incorporating immune checkpoint inhibitors into CAR-T cells [45], may be the new frontier in enhancing the tumor elimination efficacy of CAR-T cells for EOC patients.

## 3. Strategies Targeting Immunosuppression in the TME

Regulatory T cells (Tregs) participate in the establishment of the immunosuppressive TME, attenuating antitumor immunity. Neoplastic cells and TAMs produce CCL22 that mediates Treg tumor infiltration, a mechanism that could be blocked to decrease immunosuppression and enhance antitumoral immunity [46,47]. T cells transduced to express chemokine receptors matching the TME chemokines can improve tumor homing after ACT, as shown by improved migration of tumor ascites lymphocytes to the EOC microenvironment by T-cell CXCR2 transduction [48].

Highly immunosuppressive MDSCs are attractive targets to enhance the efficacy of cancer immunotherapy. Immunizing mice with microparticles containing TLR9 and NOD-2 ligands (MIS416), followed by anti-CD11b administration, was shown to abrogate the immunosuppressive capacity of MDSCs in the ID8 murine model of EOC [49]. This treatment significantly prolonged survival, highlighting the need for more immunotherapies targeting innate immunity within the TME [49]. However, there are still many unknowns concerning the different MDSC phenotypes and levels in tumor tissue, peripheral blood, and/or ascites fluid, and how their presence influences the TME [50].

TGF-β plays a key role in EOC TME by preventing antitumoral T cell responses. Recent studies have identified stromal TGF-β signaling as a determinant of immune exclusion [51,52]. In a model of colorectal cancer, Tauriello and colleagues recently showed that TGF-β inhibition prevents tumor metastasis by increasing cytotoxic T-cell responses [53]. Thus, blocking TGF-β production along with immunotherapy could be a promising pro-immunogenic approach in EOC by promoting strong T cell infiltration and antitumoral immunity [51,54].

A recent study found that the accumulation of effector memory CD8+ T cells (T_EM_) in EOC ascites was mediated by TAM-derived CXCL9. This accumulation of CD8+ T cells correlated with increased patient survival. However, ascites-derived factors can suppress T_EM_ effector functions through the production of IFNγ and TNFα, and CD107a expression, shortening relapse-free survival of patients. Inducing TAMs to produce CXCL9, CXCL10, and CXCL-11 chemokines may potentially be therapeutic [55]. COX inhibitors also enhance chemokine release, suggesting their use in combinatorial strategies to increase TILs within the EOC TME [56].

Increased expression of immune checkpoint molecules on cancer cells, antigen-presenting cells (APCs), and T cells in the EOC TME leads to immunosuppression upon binding of their corresponding receptor/ligand, effectively putting the brakes on CD8+ effector T and NK cells. Blockage of co-inhibitory molecules has now been exploited in many cancer types to increase pre-existing patient antitumoral responses (reviewed in the literature [57]). These co-inhibitory molecules include cytotoxic T-lymphocyte associated protein 4 (CTLA-4; on T cells to control T cell activation, and binds to CD80 or CD86 on APCs or tumor cells), lymphocyte-activation gene 3 (LAG-3; on T and NK cells, and binds to MHC-II and LSECtin on APCs or tumor cells [58]), programmed cell death protein 1 (PD-1; on activated T, B, and NK cells, and binds to PD-L1 or PD-L2 on APCs or tumor cells), and PD-L1 (on APC or tumor cells, and binds to PD-1 on T cells or CD80 on APC or tumor cells).

Anti-PD-1 and anti-LAG-3 synergized in the ID8-OT-I murine model to prolong survival, reduce tumor burden, and reduce Tregs, while increasing CD4+ and CD8+ TILs [59]. Therefore, blockade of immune inhibitory molecules, such as LAG-3, T cell immunoglobulin and mucin domain 3 (TIM-3); or T cell immunoreceptor with Ig and ITIM domains (TIGIT); as well as fibrinogen-like protein 2 (FGL-2) [60,61,62], may synergize with PD-1/PD-L1 to target multiple cell types and more potently relieve immunosuppression [58,63]. Interestingly, Lin and colleagues found that the efficacy of anti-PD-L1 therapy was unaltered by tumor cell expression of PD-L1 and instead, DC and macrophage PD-L1 expression was likely to underlie response [64]. Thus, more studies need to explore how these immunotherapies abrogate immunosuppression and the cell types underlying responses.

Beyond the immunosuppressive cytokine milieu present in the TME, antigen persistence also contributes to long-lasting T cell exhaustion (reviewed in the literature [65]), in part through increasing PD-1 expression and through de novo methylation of effector function genes [66]. Thus, targeting aberrant methylation using epigenetic modifiers such as the DNA methyltransferase inhibitor, azacytidine, has offered promising results in rejuvenating T cell responses [66]. Further, azacytidine induces the expression of antigen processing machinery and activates the interferon response in cancer cells by inducing viral mimicry through the MDA5/MAVS/IRF7 pathway [67,68]. However, azacytidine can also upregulate PD-L1 on cancer cells [69], and thus shift the balance from antitumor immunity toward immunosuppression. Combinatorial approaches with epigenetic therapies and immune checkpoint blockade (anti-PD-1) have been employed and shown to enhance CD8+ T and NK cell recruitment and function, and overall survival in murine models [66,70].

## 4. Increasing Tumor Immunogenicity

### 4.1. Chemotherapy

Chemotherapeutic agents used in standard EOC treatment can induce an immunogenic cell death of cancer cells by the release of danger signals known as damage-associated molecular patterns (DAMPs) [71,72]. Memory T cells derived from peripheral blood mononuclear cells from EOC patients after cytoreductive surgery and platinum and taxane chemotherapy recognized antigens associated with apoptotic EOC cells, and their presence was correlated with prolonged survival [73]. Responders displayed significant IFNγ or IL-17 functions by both CD4+ and CD8+ T cells in response to apoptotic EOC antigens [73]. Exposure to platinum-based drugs can disrupt the STAT6-mediated immunosuppression in the TME by decreasing the expression of PD-L2 on both human DCs and tumor cells, enhancing antigen-specific proliferation and Th1 cytokine secretion, as well as increasing tumor T cell recognition [74,75]. CD8+ T-cell function is not permanently suppressed in advanced EOC and successful carboplatin/paclitaxel chemotherapy is associated with improved antigen-specific T cell reactivity [76].

Paclitaxel has been previously reported to be a ligand to Toll-like receptor 4 (TLR4), normally found on normal and neoplastic cells, which, under ligation, significantly increase the secretion of IL-6 and IL-8 by human EOC cell lines (SKOV3, OVCAR3), abrogating paclitaxel effects on cell proliferation, and promoting tumor survival and chemoresistance [77,78,79]. MyD88 expression is more restricted to EOC cells, independent of tumor grade, and is associated with reduced progression-free and overall survival [80]. Strategies aiming to target the TLR4 signaling pathway on TLR4/MyD88(+) EOC patients may hold promise for the treatment of paclitaxel-resistant EOC. For example, atractylenolide-I (AO-I), a naturally occurring sesquiterpene lactone and TLR4-antagonizing agent, inhibits TLR4 signaling by interfering with the binding of paclitaxel to membrane TLR4, thus sensitizing the response of MyD88(+) EOC cells to paclitaxel [81]. AO-I indirectly downregulates MyD88/NF-κB signaling; reduces activation of NF-κB, Akt, and indoleamine 2,3-dioxygenase (IDO)-1; and attenuates the secretion of IL-6, TGF-β1, VEGF, and IL-17A by SKOV3 cells [82]. In another study, Peng and colleagues found that chemotherapy induces local immune suppression by increasing PD-L1 expression in ovarian tumor cells [83]. Paclitaxel treatment is able to increase CD8+ T-cell infiltration of ovarian tumors in a mouse model of ovarian cancer by upregulating MHC-I expression, as well as PD-L1 expression, in an NF-κB-dependent manner [83]. Thus, the authors showed that by combining paclitaxel treatment with anti-PD-L1 or anti-PD1 antibodies, the immunosuppressive TME is attenuated, enabling the achievement of maximal antitumoral responses and increasing survival of ovarian tumor-bearing mice [83].

Agents such as cyclophosphamide can also be used in combination with immune checkpoint inhibitors, such as anti-PD-1, to gain therapeutic synergy by decreasing Treg infiltration and stimulating the generation of CD8+ TILs [84]. Gemcitabine chemotherapy combined with CTLA-4 blockade results in a potent antitumor immune response that is CD4+ and CD8+ T-cell dependent [85]. Oxaliplatin treatment can enhance susceptibility of human EOC cells to NK cell-mediated cytolysis by inducing the production of type I IFN and chemokines, and enhance MHC class I-related chains A/B, UL16-binding protein (ULBP)-3, CD155, and TNF-related apoptosis-inducing ligand (TRAIL)-R1/R2 expression [86].

Chemotherapy possesses immunomodulatory properties by augmenting pre-existing TIL responses in high-grade serous ovarian cancer (HGSC) patients; however, this increase fails to confer significant prognostic benefit [87]. In contrast, platinum-resistant EOC has been shown to generate poor immunologic responses [88]. Several studies have assessed the clinical and cost-effectiveness of different combined chemotherapeutic approaches for advanced recurrent or refractory EOC [89,90]. By combining standard chemotherapeutic agents with immunotherapies, chemotherapy-induced apoptosis can be exploited as an adjuvant to synergistically enhance antitumoral immunity.

EOC-derived ascites offers accessible and plentiful tumor tissue to identify and target molecules that shape the TME. Both the cellular and fluid compartments allow for the investigation of prognostic and predictive biomarkers, pharmacodynamic markers, and molecular profiling analysis [91]. Moreover, ascites may be a useful tool to reveal the immune status of the TME within the peritoneal cavity. For example, IL-6 is enriched in the malignant EOC ascites, enhancing the invasive properties of EOC cells [92]. IL-6 levels are elevated in recurrent compared with primary advanced EOC [93], and because the IL-6R/STAT3/miR-204 feedback loop contributes to cisplatin resistance of EOC [94], targeting IL-6 or IL6R with neutralizing antibodies could increase EOC sensitivity to cisplatin. IL-6 levels can also be modulated using NF-κB inhibitors, like dehydroxymethylepoxyquinomicin (DHMEQ), as showed by Nishio and colleagues, who evaluated the effects of DHMEQ in vitro on human EOC cells and macrophages [95]. DHMEQ was able to inhibit the production of IL-6 and IL-8 by EOC cell lines and enable the release of immunosuppression of human DCs and macrophages incubated with culture supernatant of EOC pretreated cells. In vivo studies in nude mice implanted with human EOC cells showed a reduction of arginase expression and tumor accumulation of MDSCs, demonstrating the important role of NF-κB in maintaining EOC TME immunosuppression [95]. Given NF-κB is a pleiotropic transcription factor and also possesses anti-inflammatory properties [96], further studies are needed to evaluate the therapeutic potential of DHMEQ in EOC in immunocompetent hosts to better evaluate its impact on all the components of the EOC TME.

### 4.2. Oncolytic Viruses

Oncolytic viruses (OV) can be engineered for transgene expression to enhance their tumor specificity, safety, drug susceptibility, immunogenicity, and oncolytic potency [97]. They can be administered locally or intravenously and spread to the tumor and metastases [98,99]. OVs trigger at least two modes of cancer cell killing: direct oncolysis of infected cells or indirect cell death elicited by the host immune system [97,100]. These phenomena allow the release of viral antigens, DAMPs, and TAAs, which, under a proper inflammatory milieu, enable their recognition and phagocytosis by immune cells, such as macrophages and DCs, to eventually activate T cells in the draining lymph nodes [97,100,101]. OV have tumor specificity because, unlike malignant cells, healthy cells can respond to infection by inducing antiviral IFNs, though tumor heterogeneity in IFN expression has been identified as means of OV resistance [102].

OVs are a new class of immunotherapy that have shown promising results in clinical studies, leading to the approval of the first OV, talimogene laherparepvec (Imlygic^®^, Amgen, Thousand Oaks, CA, USA) T-VEC, for treatment of metastatic melanoma [103,104,105,106]. Notably, OVs have shown synergy when combined with other immunotherapies, such as checkpoint inhibitor antibodies [103,104,106]. IL-12 expressing oncolytic herpes simplex virus was shown to promote eradication of both murine and human ovarian cancer cell lines and promote TAA-specific CD8+ T-cell responses in the peritoneal cavity and omentum, leading to reduced peritoneal metastasis and improved survival in the mouse model tgMISIIRTAg [107]. Thus, the use of OV immunotherapy alone or combined with approaches to increase immunostimulatory or immunogenic responses offers promising strategies for investigation as novel treatments for EOC patients.

In addition to cancer cells, OVs can infect and lyse CAFs [108] and endothelial cells in the TME [109,110], leading to the destruction of these cells and immune infiltration [111]. At present, several viruses are under active investigation in preclinical [112,113,114,115,116,117] and clinical trials (NCT02028117, NCT00408590, NCT02759588, NCT02068794, NCT03225989, NCT01199263, NCT02285816) as potential therapies for various cancers, including EOC, as well as in combinatorial strategies with other immunotherapies such as checkpoint inhibitors.

**Figure 1:**
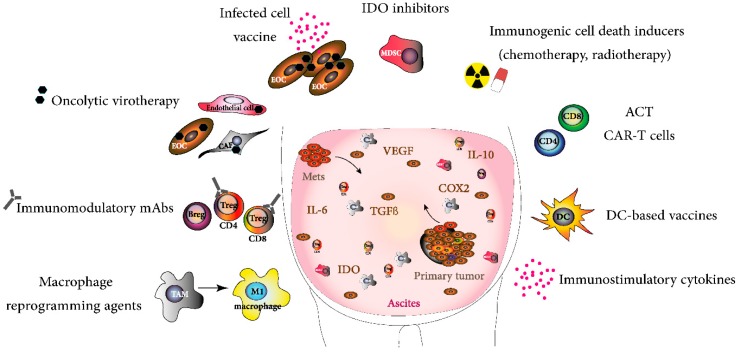
Emerging ovarian cancer immunotherapies. T cell infiltrated EOCs (>50% EOCs [120] can be targeted with therapies such as ACT, CARs cells, co-stimulatory mAbs [121] (like anti-CD137), oncolytic virotherapies, and DC-based vaccines [122], aiming to increase the effector functions of the pre-existing antitumoral immunity. Conversely, strategies aiming to decrease the highly immunosuppressive TME [checkpoint blockers mAbs (anti-PD-L1), IDO inhibitors [123], etc.] can be exploited for ‘cold’ tumors and/or for advanced stages EOC, to decrease the immunosuppressive functions of MDSCs, TAMs, and Tregs. Radiotherapy and chemotherapy are immunogenic cell death inducers increasing the release of TAAs in the TME, thus augmenting the NK-cell mediated killing, the incidence of TAA presentation by APCs, and eventually T cell priming. Also, chemotherapy can target MDSCs (gemcitabine [124], 5-fluorouracil [125]). EOC cells and TAMs can be targeted with trabectedin, which inhibits CCL2 production and decreases monocyte recruitment in tumors [126]. OVs can infect tumor cells, as well as CAFs and endothelial cells, thereby helping to decrease their immunosuppressive action in the TME. Many approaches can be combined, such as administration of costimulatory cytokines (IL-2, IL-7, IL-15 and IL-21) along with approaches such as CARs, OVs, and ACT. Tumor cells derived from ascites can be exploited for the production of infected cell vaccines with OVs delivering IL-12. Simultaneous targeting of CD137 and PD-1 [127] or TIM-3 [128] with mAbs along with cisplatin treatment [129] can achieve significant antitumoral responses. Adoptive cell transfer (ACT), cancer-associated fibroblasts (CAFs), chimeric antigen receptor (CAR), monoclonal antibodies (mAbs), natural killer (NK), myeloid-derived suppressor cell (MDSC), tumor associated macrophage (TAM), regulatory T cells (Tregs), tumor associated antigen (TAA), antigen presenting cell (APC), oncolytic virus (OV), programmed cell death 1 (PD-1), T-cell immunoglobulin and mucin domain 3 (TIM-3), metastasis (Mets).

A promising new strategy for cancer immunotherapy is to exploit autologous tumor cells as carriers of viruses to the tumor niche [118]. Such oncolytic vaccine platforms consisting of tumor cells infected with OV have been shown to be a favorable strategy in murine models of melanoma and other solid cancers [101,103]. An infected cell vaccine (ICV) platform was developed using irradiated autologous tumor cells infected with oncolytic Maraba (MG1) virus that is engineered to express the immune stimulatory cytokine IL-12 [119]. When delivered directly into the peritoneal cavity in a model of peritoneal carcinomatosis, the vaccine promoted the migration of IFNγ-secreting NK cells, decreased tumor burden, and improved survival. Importantly, the enhanced NK-cell cytotoxicity and migratory capacity driven by ICV-MG1-IL12 was also observed in human lymphocytes exposed to human tumor cell lines infected with MG1-IL12, highlighting the benefit of this approach in patients with abdominal cancers [119]. MG1 is currently being evaluated in phase I/II clinical trials as a stand-alone therapy and in a vaccination strategy for the treatment of late-stage disseminated disease (NCT02285816, NCT02879760). Thus, by using tumor cells as virus carriers, the TME can be remodeled, making a “cold” tumor into an inflamed or “hot” tumor that could support and sustain the generation of significant antitumoral responses. This approach could be advantageous to EOC patients whose tumors have suboptimal immune infiltration and do not respond to standard therapies. Additional studies are needed to assess the conditions (TME quality, tumor grade, etc.) under which this approach could benefit those EOC patients, as well as to determine if this rationale could be exploited with tumor cells derived from EOC ascites. Figure 1 summarizes some emerging EOC immunotherapies.

## 5. Preclinical Models for Ovarian Cancer Immunotherapy

The ovarian cancer field has accelerated rapidly since the discovery that ovarian cancer is not one disease, but exists as numerous subtypes that behave differently. For the most common EOC subtype, HGSC, we have only recently begun to appreciate the role of the fallopian tube secretory epithelium (murine oviductal epithelium) as one of the origins of HGSC. These discoveries led researchers to focus on modeling ovarian cancer after specific subtypes or origin(s) of disease, leading to a narrow characterization of many of these preclinical models in relation to their origin and common genomic alterations with HGSC. Commonly studied features include growth rate, tumorigenic potential, immunohistochemical markers, DNA mutations, BRCAness, RNA expression, and copy number variation, with the results correlated to The Cancer Genome Atlas (TCGA) or other large datasets on HGSC. Since the emergence of immunotherapies as promising agents for ovarian cancer treatment, we have a rich reservoir of human and murine-derived ovarian cancer models that have limited characterization for the features that we have come to appreciate as important indicators of immunotherapeutic efficacy. Such features include MHC status and PD-L1/2 expression on cancer cells, total mutational and neoantigen burden, ascites composition, hot versus cold immune landscape, and the contribution of tumor stroma (cancer-associated fibroblasts) to tumor immunosuppression. Given that the current clinical efficacy of immune checkpoint blockade for HGSC ranges from 9.7 to 15% [35], preclinical models will be of great importance as we seek better indicators of response, as well as new immunotherapy development. In this section, we summarize the current knowledge of ovarian cancer models and highlight the models that may be untapped resources for the immunotherapy field.

### 5.1. Syngeneic Murine Models

A syngeneic model is defined by its immunological compatibility such that the host does not reject either the outgrowth or transplant of cancer cells in immunocompetent animals. We have divided syngeneic models into spontaneously occurring and genetically engineered models, as described below.

#### 5.1.1. Spontaneously Transformed Syngeneic Models

The only two non-human animals that are known to spontaneously develop EOC are the egg-laying hen and the jaguar (Table 2). Up to 35% of egg-laying hens develop EOC with similar risk factors to humans such as age and ovulation number, reviewed in the literature [130]. The hen model has yet to be used for immunotherapy development, but has features that make it an exciting candidate for future use. The first evidence that spontaneous hen tumors are immunogenic was the observation of mesothelin auto-antibodies and mRNA in 44% of hens harboring tumors [131]. Serous histology hen tumors contain the most TILs characterized by T and B cell infiltration [132], and highly express immunosuppressive ILT3, which functions to limit T cell proliferation and differentiation, suggesting perturbed antitumor adaptive immunity within the TME [133]. Barua and colleagues showed elevated expression of DR6, a known inhibitor of DC function, with increasing stage of disease, suggesting perturbed innate immunity, as well as adaptive immunity [134]. This group then showed that increased immune infiltration in late-stage disease is restricted to the tumor stroma, while intratumoral immune infiltration largely decreases with the stage, indicating that late-stage tumors acquire mechanisms to limit immune trafficking [135]. These studies support the presence of immunosuppression within the hen TME and highlight the hen model’s promise for immunotherapy development. Profiling immune checkpoint expression, total mutation, and neoantigen burden in hen tumors would be an exciting addition to the hen model dataset.

Interestingly, 40% of captive jaguars develop ovarian carcinoma with non-synonymous mutations in *BRCA1* [136,137]. The endangered nature of this species prevents its use as an ovarian cancer model, though immunotherapies that enhance survival of patients with *BRCA1*-associated cancers could later play a role in the conservation of this species.

The ID8 and STOSE models (in the C57BL/6 and FVB/N strains, respectively) are two incidences of spontaneous transformation of primary murine ovarian surface epithelial cell cultures. Both of these models share similar epithelial markers [cytokeratin(+), WT1(+), inhibin(−)], growth rates, expression profiles similar to human HGSC, and tumorigenicity in syngeneic xenografts [138,139], and they both form malignant ascites and disseminated disease following orthotopic intrabursal injection [138,140]. The ID8 model, established by Roby and colleagues in 2000, has been the most commonly used model for immunotherapy development based on its established characterization and reliability in forming syngeneic tumors. Peritoneal tumors generated by IP injection of ID8 cells develop a complex microenvironment with SMA+ fibroblasts, CD3+ T cells, CD68+ macrophages, and neo-vasculature [140]. ID8 cells have been employed in the development of epigenetic modifiers, immune checkpoint inhibitor and oncolytic virus studies, DC and microparticle vaccines, and numerous emerging immunotherapies [112,129,141,142,143,144].

Antibody monotherapy has shown little promise in the ID8 model, as neither immune checkpoint inhibitors (anti-PD-1, anti-CTLA-4) or activating antibodies (anti-OX40, anti-CD137) used as monotherapies had any impact on survival [129,142]. Two studies using combination antibody immunotherapy, anti-PD-1 and -OX40 [142] or anti-PD-1 and -CTLA-4 and -CD137 [129] have shown prolonged survival of ID8 tumor-bearing mice and a shift from a CD4+ T helper 2 (Th2) cell milieu to an antitumor Th1 response characterized by an increased ratio of CD8+ and CD4+ T cells over immunosuppressive CD4+FOXP3+ Tregs, and a reduction in CD11b+Gr-1+ MDSCs. In both studies, antibodies were administered within 15 days of ID8 injection, representing early-stage disease before robust tumor formation and ascites develops. It remains to be determined if these therapies promote regression of late-stage disease in the presence of ascites. Turner and colleagues reported enhanced MHC class II expression in subcutaneous ID8 tumors and restricted tumor growth with a combined epigenetic therapy using a histone deacetylase inhibitor (entinostat) and DNA methyltransferase inhibitor (azacytidine). Furthermore, azacytidine enhances recruitment of CD8 T and NK cells in the ID8 model, and shows synergy with anti-PD-1 checkpoint blockade, offering an alternative approach to modify the immune landscape of the TME [70,144].

Numerous OV platforms have been tested in the ID8 model including reovirus, vaccinia, myxoma, vesicular stomatitis, and herpes simplex viruses [109,112,141,143,145]. Many of these OV platforms have been shown to prolong survival, enhance CD8+ T-cell infiltration, and reduce immunosuppression. Oncolytic vaccinia virus encoding a CXCR4 antagonist helped prevent peritoneal spread and reduced Treg recruitment in ID8 tumors [112]. Although promising, this monotherapy failed to cure the ID8 model, highlighting the need for combinatorial therapies [112]. Synergy was observed when myxoma virus was administered prior to cisplatin treatment in an IP ID8 model, generating a T-cell response that could recognize TAAs from ID8 cell lysates [143]. A recent study by Liu and colleagues reported synergy between anti-PD-1 antibody therapy and oncolytic vaccinia virus in the ID8 model [146], opening the door to combining oncolytic platforms and immune checkpoint inhibition or novel antibody therapies.

Among the studies using ID8 cells, none have identified a full curative therapy that generates a robust memory response that can protect against ID8 cell re-challenge, a sought-after goal of immunotherapies. This could be because of the poor immunogenicity of ID8 cells; out of their mutational burden of 92 somatic mutations, only 17 are predicted to generate transcribed neoantigens. Upon vaccination with synthetic peptides carrying these 17 mutations, none induced a neoantigen-specific T-cell response, indicating that they likely do not yield MHC presented epitopes [147]. The use of modified ID8 cell lines may better phenocopy human HGSC, given that parental ID8 cells have a relatively low mutational burden compared with human HGSC [147].

One of the first modifications to ID8 cells was the stable expression of beta-defensin, *Defb29*, and *Vegf-A* yielding ID8-Defb29/Veg-A cells that had increased pro-tumor DC recruitment, neovasculature, and a more aggressive phenotype with reduced survival compared with parental ID8 cells [148]. ID8-Defb29/Veg-A derived tumors are good models for DC dysfunction and recently, Cubillos-Ruiz and colleagues identified the role of the endoplasmic reticulum stress sensor XBP1 in mediating DC dysfunction in this model [149]. A second modification was the addition of the ovalbumin (OVA) peptide, ID8-OVA, a useful tool to assess antitumoral responses mediated by OT-I CD8+ T cells or OT-II CD4+ T cells derived from the transgenic mouse models OT-I and OT-II, where the TCRs were designed to specifically recognize OVA peptides in the context of H2Kb and I-A b, respectively [141,150]. Using a reovirus platform, Chiang and colleagues showed prolonged survival, enhanced expression of MHC class I antigen presentation machinery (beta-2-microglobulin, TAP-1, and TAP-2), reduction of MDSCs and Tregs, and enhanced DC-activation of OVA-specific CD8+ T cells in the ID8-OVA model [141].

One of the notable weaknesses of the ID8 model is that it does not contain a *Trp53* mutation, which is characteristic of 94% of human HGSC [151]. Walton and colleagues generated ID8 cells with both a *Trp53* and *Brca2* mutation using CRISPR-Cas9 [152,153]. ID8-*Trp53*−/− tumors had increased MDSCs recruitment, possibly through increased CCL2 expression. With the addition of *Brca2* deletion, the tumors gained intraepithelial lymphoid aggregates, characteristic of hereditary human HGSC (~9% of cases), making this model relevant to the study of *BRCA*-associated HGSC [154]. Given the observed increase in mutational burden in HGSC of BRCA mutation carriers [155], it would be interesting to profile neoantigen burden and TAA-specific T-cell responses in ID8-Trp53-Brca2 cells, as well as ID8-Defb29/Vegf-A, to further enhance their relevance to HGSC. These modified ID8 cell lines may offer more relevant models for ovarian cancer immunotherapy as they better phenocopy the TME found in human HGSC. Roberts and colleagues have also published a spontaneously transformed murine ovarian surface epithelial cell line, MOSE-L cells, which were highly proliferative, expressed epithelial markers, and are tumorigenic in syngeneic C57BL/6 mice, though uptake of this model has been sparse, likely because of the well-established characterization of the ID8 model [156].

In 2014, our group published the second spontaneously transformed syngeneic model of HGSC-like cancer, the STOSE model, which reliably generates tumors in syngeneic FVB/N mice. Our initial characterization profiled the growth rate, genomic instability, and immunohistochemical markers relevant to human HGSC [138]. Recently, we have expanded the characterization of the STOSE model by profiling the immune landscape of orthotopic intrabursal-derived STOSE tumors and have found STOSE tumors have increased T-cell infiltration and a larger CD4+ Treg population than ID8 tumors (data not shown), suggesting STOSE tumors contain a T-cell-rich or ‘hot’ TME. In contrast, orthotopic ID8 tumors generate a more myeloid-rich or ‘cold’ TME. Given their susceptibility to oncolytic virus infection in vitro [107], it will be important to compare the efficacy of immunotherapeutic approaches in these two models, which generate tumors in different murine strains and have contrasting immune landscapes within the TME, and may better reflect the heterogeneity of HGSC seen in the clinic. Characterization of the STOSE model for copy number and mutational and neoantigen burden must be done to assess the utility of the STOSE model.

Both ID8 and STOSE models are derived from the ovarian surface epithelium. Given the contribution of the fallopian tube epithelium to human HGSC, spontaneous and transplantable syngeneic models derived from murine oviductal epithelium are much needed. Endsley and colleagues described spontaneously transformed murine oviductal epithelial cells derived from CD1 mice that exhibited features of transformation, but only generated subcutaneous tumors in athymic nude mice, limiting their use as a syngeneic model for cancer immunotherapy studies [157]. The addition of PTEN loss in these cells resulted in the first and, currently, the only syngeneic model of fallopian tube-derived EOC [158].

#### 5.1.2. Genetically Engineered Mouse Models (GEMM)

The majority of GEMM models were generated to improve our understanding of the origin(s) of ovarian cancer. Consequently, most of the characterization of these models has placed primary emphasis on identifying the location of early lesions and an immunohistochemical panel assessing positivity for PAX8, P53, WT1, and cytokeratins with a lack of inhibin and calretinin staining. With emerging immunotherapies targeting the TME, characterization of the majority of GEMM models has been too narrow to assess their use for testing immunotherapies, although many models may be ideal because of their shared genomic alterations and TME complexity that phenocopy HGSC. The various GEMM models of ovarian cancer have been comprehensively reviewed [159,160]. Here, we discuss some important considerations in using GEMM models for immunotherapy development.

Many models have been made using oncogenic simian-virus 40 T-antigen (SV40TAg) driven from the ovarian or oviductal epithelium (Table 3). These models include the TgMISIIRTAg model, which drives SV40TAg expression from the *MISIIR* gene, leading to ovarian tumor development in 50% of mice at 6–13 weeks of age [161]. The use of this model has revealed a synergistic effect of the viral sensitizer colchicine and vaccinia virotherapy [109]. Epigenetic combination therapy, entinostat and azacytidine, was shown to enhance MHC class II expression in TgMISIIRTAg tumors [144]. The TgCAG-LS-TAg model that drives SV40TAg from the chicken β-actin promoter was used to show that estrogen can accelerate EOC development, though an immune basis for this acceleration was not explored [162]. Another model used the oviduct-specific gene, *Ovgp1*, to drive SV40TAg expression generating tumors derived from oviductal epithelial cells [163]. Although these results showed promise, the use of SV40TAg for immunotherapy studies should proceed with caution as SV40TAg is both an oncogenic driver and a dominant immunogen. Schietinger and colleagues designed a sophisticated experiment in which SV40TAg-specific T cells (TCR_SV40-1_) and OT-I T cells were co-injected into liver tumor-bearing ASTxAlb–Cre mice that constitutively express SV40TAg. Only TCR_SV40-1_ cells became dysfunctional in the presence of cognate SV40TAg with enhanced expression of PD-1, TIM-3, LAG-3, and 2B4, while OT-I T cells maintained their functional expression of IFNγ and TNFα [164]. This showed that SV40TAg expression strongly inhibited effective T cell responses with little contribution from the immunosuppressive TME, because OT-I T cells maintained their functional phenotype within the TME. Thus, a strong clonal response to a persistent dominant antigen was enough to reduce the influence of the TME, which does not reflect the normal contribution of the TME in suppressing antitumor immunity in HGSC (reviewed in the literature [165]). Further, McGranahan and colleagues recently showed that effective cancer immunotherapy goes beyond total mutational burden and requires a clonal neoantigen T-cell response [166]. Patients who exhibit increased intratumoral neoantigen heterogeneity (subclonality) have reduced clinical benefit to immune checkpoint inhibition. They further showed that chemotherapy can induce neoantigen heterogeneity [166]. Thus, using models with a dominant antigen, such as SV40TAg, which can abrogate the effects of the TME, could confound the interpretation of an immunotherapy’s efficacy. Models that have neoantigen heterogeneity or lack authentic neoantigens (like the ID8 cells) may better phenocopy HGSC, as only 12% of HGSC are likely to express ≥1 neoantigen [147].

Given that 94% of HGSCs possess *TP53* mutations, with 35% of tumors expressing high levels of TP53 and 62% expressing little to no detectable TP53 [151], immunotherapies should be tested in models that represent both high and low p53 expression. Multiple GEMMs have either Tr*p53* knockout or *Trp53* mutation, driven from both the ovarian and oviductal epithelium [167,168,169,170]. HGSCs with mutant *TP53* have higher PD-L1 expression than tumors with wild-type *TP53*, indicating a role for mutant TP53 in modulating the TME, though the exact *TP53* mutations were not specified [171]. The effect of *TP53* loss was further corroborated by Son and colleagues who showed *p53* loss enhanced pro-inflammatory cytokine (CXCL1, CXCL2, CXCL3, and CXCL8; and TNFα) expression in HGSC [172]. More studies are needed to elucidate the effects of specific *TP53* mutations on the ovarian cancer TME.

Interestingly, hereditary *BRCA1/2* mutations lead to higher mutational burden and neoantigen burden that correlates with improved survival, increased TILs, and increased PD-1/PD-L1 expression, indicating that these tumors may especially benefit from immune checkpoint inhibition [155,171]. Perets and colleagues generated GEMMs with doxycycline-inducible Cre-recombinase mediated deletion of *Brca1* or *2* and *Pten*, and Tr*p53* loss or mutation, driven from the oviductal epithelium-specific *Pax8* promoter. All combinations yielded HGSC-like tumors with high mutational burden and genomic alterations similar to the TCGA dataset on ovarian carcinoma such as c-myc amplification [169]. Similarly, Zhai and colleagues characterized a model of tamoxifen-inducible deletion of *Brca1*, *Pten*, *Rb1*, and *Nf1* driven from the *Ovgp1* promoter, which generated serous-tubal intraepithelial carcinomas that progressed to HGSC. They also characterized a similar model with deletion of *Brca1*, *Pten*, and p53 that also developed precursor lesions and HGSC, but with a mixed tumor phenotype with mucinous metaplasia [173]. These models have numerous features relevant to human disease and profiling the immune landscape and mutational and neoantigen burden would be exciting additions to extend the use of these models into the realm of cancer immunotherapy.

One GEMM study that profiled the TME was done by Budiu and colleagues, using mice that express human MUC1 from the endogenous promoter that were then crossed with mice containing conditional alleles for *Pten* deletion and activation of KrasG12D [174]. MUC1 is overexpressed in 75–90% of human EOC and, interestingly, tumor-associated MUC1 is more immunogenic because of the loss of glycosylation, revealing epitopes that can be targeted by antibodies and specific CD8+ T cell responses [175,176,177]. The MUC1KrasPTEN model generates ovarian tumors with high serum levels of human MUC1, robust CD4+FOXP3+ TILs, and dysfunctional DCs [174]. Mice that expressed human MUC1 had a larger splenic Treg population than KrasPTEN mice alone. Using a MUC1 vaccination strategy along with a type 1 DC polarizing cocktail, they were able to reduce Tregs and enhance survival in MUC1KrasPTEN tumor-bearing mice [174]. This model allows for a MUC1-directed antitumor response that could be modulated by a vaccination strategy targeting the TME. In this aspect, this model improves upon SV40TAg models where the immunogen is too dominant to see any effect of the TME modulation [164]. 

Although GEMM models enable us to better model the origins of disease and genomic alterations characteristic of HGSC, they have two weaknesses that limit their use for immunotherapy. Firstly, most GEMMs have been generated on a mixed strain background, preventing the generation of transplantable syngeneic cell lines. Secondly, although GEMMs may reproducibly generate tumors, they tend to arise over a wide course of time. The difficulty in controlling tumor onset and size in GEMMs introduces a logistical challenge for immunotherapy studies that rely on flow cytometric analysis of immune populations that are generally performed simultaneously at one point in time. In contrast, this limitation is easily overcome with transplantable syngeneic models, such as the ID8 and STOSE models, where tumor onset is uniform and controlled.

## 6. Human-Derived and Autologous Cultures

Numerous ovarian cancer cell lines have been used historically with inconsistencies in their relevance to human HGSC, particularly A2780 and SKOV3 cells, which are unlikely to represent HGSC (reviewed in the literature [185]). Recently, HGSC primary cultures have been established that have genomic alterations, TAA expression, and gene expression profiles consistent with TCGA datasets [186,187,188]. These primary cultures offer resources for TAA discovery, infectivity with oncolytic viruses, and developing methods to increase immunogenicity. The major weakness of using primary cultures is that tumorigenesis can only be studied in xenografts using immunodeficient mice that fail to develop a complex TME with the immune subsets seen in patient tumors.

Ovarian cancers are rich resources for easily accessible cancer and immune cells from ascites fluid. Ascites fluid is remarkably immunosuppressive, containing high levels of Tregs and MDSCs [47,143]. Ascites have been a source for NK cells, where ex vivo expansion restored their cytotoxicity against autologous CD45-EpCAM+ cells [189]. Nounamo and colleagues showed that myxoma virus can prevent the secretion of IL-10 from ascites-derived CD14+ myeloid cells, thereby providing in vitro evidence that myxoma virus may remodel the ascites microenvironment to facilitate stronger antitumor immunity [143]. An interesting approach was developed by Freedman and colleagues using an oncolytic adenovirus expressing a bispecific T cell engager (BiTE) that targets autologous CD8+ T cells to EpCAM+ ascites cells and pleural effusions. Remarkably, even in the presence of ascitic fluid, EnAdEpCAMBiTE stimulated T cell proliferation and cytotoxicity against EpCAM+ ascites cells [190]. A co-culture system has also been developed to assess the efficacy of CAR-T cell therapy by culturing dissociated primary ovarian tumors with autologous derived anti-5T4 CAR-T cells [34]. Even though the use of human samples has limitations for studying the ovarian cancer TME, they offer invaluable resources to determine the specificity of both innate and adaptive targeted immunotherapies.

## 7. Summary

Further studies about EOC TME evolution during disease and treatment are needed. Importantly, heterogeneity among metastatic and primary tumors within a single patient can coexist [191], and this heterogeneity can influence the immune cell landscape, thereby affecting prognosis and therapeutic responses. Similarly, the ascites TME can respond differently to therapy, because EOC ascites contain another complex immunosuppressive network. Therefore, the challenges of tumor heterogeneity must be considered when designing therapeutic strategies for EOC patients.

In this review, we described some of the current emerging immunotherapies that have shown promising results in animal models and other cancer types and that could be exploited in EOC. The major barrier in EOC immunotherapy is the highly immunosuppressive TME. Thus, therapies aiming to decrease immunosuppression as a first line therapy combined with immunostimulating strategies could succeed in the fight against EOC.

We have also highlighted spontaneous and GEMM syngeneic models of ovarian cancer that offer promising characteristics for use in immunotherapy research. In moving forward, it will be important to characterize many of these models for immune infiltration, neoantigen burden, TAA, and immune checkpoint expression, as well as stromal features, in order to generate meaningful data for the immunotherapy field that goes beyond survival. Models using dominant immunogens such as SV40TAg should proceed with caution and require validation in separate models. The use of ovalbumin may offer a superior model that allows for the modulation of the ovarian cancer TME. Thus far, the ID8 model has been the most widely used, but given recent findings on the weak immunogenicity of ID8 cells [147], novel therapeutics should be tested in both spontaneous and GEMM models that cover a wide range of the tumor heterogeneity seen in the clinic.

New sequencing technologies have enhanced our ability to look deeper into tumors, stroma, and TIL compartments. These studies have revealed the impact of genetic heterogeneity and epigenetic plasticity in cancer evolution during treatment (drug resistance) and clinical outcome [192,193]. At present, we know that a tumor is not a single entity determined solely by genetic alterations, but a whole complex network that affects surrounding healthy cells provoking tumorigenesis advantage. Thus, in order to achieve significant responses to eradicate neoplastic cells, TME screening (TIL composition and quality) must be considered to better assign a therapeutic strategy to a patient, especially in advanced EOC stages. The detection of key biomarkers allowing the prediction of responsiveness to an immunotherapeutic approach is also necessary to select the best strategies and combined therapies that have the maximum potential to fully eradicate cancers [1].

## Figures and Tables

**Table 1:** Main subsets of immune infiltrating cells in epithelial ovarian cancer (EOC) tumor microenvironment (TME).

Immune Cell Type	Antitumoral Function	Tumor-Promoting Function
**CD4+ Th1 cells**	Help to CTLs in tumor rejection and production of TNFα, IFNγ, and IL-2	Production of cytokines
**CD4+ Th2 cells**		Education of macrophages, production of cytokines, B cell activation
**CD4+ Treg Cells**	Suppression of inflammation (cytokines and other suppressive mechanisms)	Immunosuppression: causes IL-2 and other cytokine deprivation, production of TGFβ, IL-10, impaired activation of CTLs
**CD8+ T Cells**	Direct lysis of cancer cells and production of pro-inflammatory cytokines TNFα, IFNγ, and IL-2	FOXP3+ CTLA-4+ CD25+, convert effector CD8+ T cells into suppressor cells, suppressive function through TGF-β1
**B Cells**	Production of tumor specific antibodies, IFNγ, TAA presentation, Th1 polarization, promotes T cell expansion	Production of IL-6, IL-10, IL-35, TGFβ, CCL22, immunosuppression, T cell conversion to Tregs, promotes Th2 inhibitory responses
**Macrophages, DCs**	TAA sampling and presentation; T-cell priming; and production of IL-12 and type I IFN, lympho-attracting chemokines CXCL9, CXCL10, CXCL11	Promotes metastasis and invasion. Produces CSF-1, arginase, IL-6, IL-10, and CCL22. B7-H4+ TAMs suppress antitumoral responses.
**MDSCs**		Immunosuppression, induces Tregs differentiation, M2 TAM, cancer stemness, sphere formation, and metastasis. Defective TAA presentation. Production of arginase-1, nitric oxide, reactive oxygen and nitrogen species, prostaglandin E2, CXCL12. Deplete cysteine, induce Tregs, inhibit T-cell activation and proliferation, and attenuate the cytolytic ability of NK cells.
**NK Cells**	Direct cytotoxicity toward cancer cells and production of pro-inflammatory cytokines GM-CSF, TNFα, IFNγ, IL-2 and chemokine CCL5	

CD4+ helper T cells (Th), cytotoxic T lymphocytes (CTLs), interferon (IFN), interleukin (IL), transforming growth factor beta (TGFβ), forkhead box P3 (FoxP3), cytotoxic T-lymphocyte associated protein 4 (CTLA-4), tumor-associated antigens (TAAs), tumor-associated macrophages (TAMs), dendritic cells (DCs), colony stimulating factor 1 (CSF1), granulocyte-macrophage colony-stimulating factor (GM-CSF), myeloid-derived suppressor cells (MDSCs), natural killer cells (NKs), regulatory T cells (Tregs). See the literature [12] for details.

**Table 2:** The utility of spontaneous and syngeneic models of ovarian cancer.

Model	Genetic Engineering	Key Features of Tumor Immune Landscape	Mutation/Neoantigen Burden	Advantages	Limitations	References
Laying Hen	None	- T and B cell infiltration - Immunosuppressive DR6 and ILT3 expression	Unknown	- Shared risk factors with human disease - Tumors classified from Stage I–IV similar to HGSC - Ascites develops in later stages II–IV	- Time > 2 years for tumor development - Lack of reagents for species	[131,132,133,134,178]
Jaguar	None	- Unknown	Familial BRCA mutations	- Shared risk factors and familial BRCA mutations similar to high-risk women	- Endangered species - Lack of reagents for species	[136,137]
ID8-(original)	None	- Fully profiled - Predominant innate cell infiltration	Low	- Reliable and fast tumorigenesis - Well characterized - Develops ascites	- Lacking mutations common to human HGSC	[139,147]
ID8-Defb29/Vegf-A	Stable Defb29 and Vegf-A expression	- Robust DC infiltration	Unknown	- Dysfunctional DCs characteristic of human HGSC - Aggressive - Forms neovasculature	- Lacking mutations common to HGSC	[148,149,179,180]
ID8-OVA	Stable ovalbumin expression	- Not profiled	OVA	- Immunogenic with DC vaccination strategy - Can track T cell responses against OVA - Allow antitumoral T cell studies with transgenic mice	- OVA dominance may not reflect the nature of TAAs in HGSC	[141]
ID8-Trp53−/− Brca2−/−	*Trp53* and *Brca2* deletion	- Increased MDSCs recruitment - Develops intraepithelial lymphoid aggregates	Unknown	- Shared genomic alterations with human HGSC - Complex immune landscape similar to human HGSC	- *Trp53* deletion may not reflect biology of *TP53* mutations seen in human HGSC	[152,153]
ID8-NGL	NF-kappaB-dependent GFP/luciferase expression	- M2 macrophages dominant immune cell type in ascites	Unknown	- Track tumor cells in vivo - Assess role of NF-kappaB on immune function	- Ascites fluid interferes with luciferase signal - Lacking mutations common to human HGSC - Luciferase can act as a neoantigen	[181,182]
STOSE	None	- Not profiled - Predominant Treg infiltration	Unknown	- Reliable and fast tumorigenesis - Different mouse strain than ID8 model - Give rise to T cell inflamed tumors - Develops ascites	- Lacking mutations common to human HGSC	[138]

High-grade serous ovarian cancer (HGSC), ovalbumin (OVA).

**Table 3:** The utility of genetically engineered mouse models (GEMM) of ovarian cancers.

Model	Genetic Engineering	Key Features of Tumor Immune Landscape	Mutation/Neoantigen Burden	Advantages	Disadvantages	References
TgMISIIRTAg	SV40TAg driven from reproductive tract-specific MISIIR (*Amhr2*) promoter during development	Epigenetic modifiers enhance MHCII expression on cancer cells	Unknown	- Forms ascites	- SV40TAg - Slow tumor development (6–13 weeks) - Non-inducible tumorigenesis	[144,161]
TgCAG-LS-TAg	SV40TAg with lox-stop cassette driven from ubiquitous CAG promoter *	Unknown	Unknown	- Ascites develops in a subset of mice - Inducible SV40TAg	- SV40TAg - Slow tumor development—>22 weeks - Surgical administration of Ad-Cre	[162]
mogp-TAg	SV40TAg driven from oviduct-specific *Ovgp1* promoter	Unknown	Unknown	- Oviduct tumor origin	- SV40TAg - Non-inducible tumorigenesis - Slow tumor development (>6 weeks) - Fails to develop ascites	[163,183]
TgK18-GT121-Brca-Trp53	Inducible SV40TAg and either *Trp53*−/− or *Trp53*mut and *Brca1* or *2* deletions driven from epithelial specific cytokeratin 18 expression *	Unknown	Unknown	- R172H *Trp53* mutation that phenocopies human R175H *TP53* mutation - Inducible SV40TAg	- SV40TAg - Surgical administration of Ad-Cre	[170]
Trp53loxP/loxP-Rb1loxP/loxP	Inducible deletion of *Trp53* and *Rb1* *	Unknown	Unknown	- Inducible gene deletions - Genomic alterations similar to human HGSC	- *Trp53* deletion may not reflect biology of all *TP53* mutations seen in HGSC - Slow tumor development (median survival 227 days)	[167,168]
Pax8-Cre-Brca1(2) −/−; Trp53mut(−/−);Pten −/− *	Doxycyline inducible Cre-mediated deletion of *Brca*, *Pten*, and *Trp53*, driven from oviduct-specific *Pax8* promoter.	Unknown	Copy number alterations similar to HGSC, Neoantigen and mutation burden unknown	- Inducible gene deletions from oviduct origin - Genomic alterations similar to human HGSC - Models with both *Trp53* deletion and mutation	- Fails to develop ascites - *Pten* deletion induces endometrial lesions	[169]
Ovgp1-iCre-ERT2 + tumor suppressor genes	Conditional deletion of *Brca1*, *Pten*, *Rb1*, and *Nf1* (BPRN mice) or *Brca1*, *Pten*, and *p53* (BPP mice), driven from the oviduct-specific *Ovgp1* promoter	Unknown	Unknown	- Inducible gene deletions from oviduct origin - Genomic alterations similar to human HGSC - Models with both Trp53 deletion and mutation	- Ascites only in 12% of mice - BPP mice develop a mixed tumor phenotype with mucinous metaplasia	[173]
MUC1KrasPTEN	Constitutive expression of human MUC1 and inducible oncogenic KRAS^G12D^ and *Pten* deletion. *	Robust Tregs among TILs and dysfunctional DCs	unknown	- Expression of human TAA MUC1 - Inducible activation of KRAS^G12D^ and deletion of *Pten* - Tumor development from both ovary and fallopian tube - Shared genomic alterations with endometroid ovarian cancer	- Surgical administration of Ad-Cre - Lacking common genetic alterations with human HGSC	[174,184]

* Model tissue-specificity governed by the site of administration of adenovirus expressing Cre recombinase (Ad-Cre). Tumor infiltrating lymphocytes (TIL).
